# Anxiety during Radiation Therapy: A Prospective Randomized Controlled Trial Evaluating a Specific One-on-One Procedure Announcement Provided by a Radiation Therapist

**DOI:** 10.3390/cancers13112572

**Published:** 2021-05-24

**Authors:** Delphine Antoni, Céline Vigneron, Jean-Baptiste Clavier, Sébastien Guihard, Michel Velten, Georges Noel

**Affiliations:** 1 Department of Radiotherapy, ICANS, Institut de Cancérologie Strasbourg Europe, Paul Strauss Comprehensive Cancer Center, 17 rue Albert Calmette, 67200 Strasbourg, France; c.vigneron@icans.eu (C.V.); jb.clavier@icans.eu (J.-B.C.); s.guihard@icans.eu (S.G.); g.noel@icans.eu (G.N.); 2 Clinical Research Department, Institut de Cancérologie Strasbourg Europe, 17 rue Albert Calmette, 67200 Strasbourg, France; m.velten@icans.eu

**Keywords:** one-on-one announcement, radiation therapist, anxiety, breast cancer

## Abstract

**Simple Summary:**

What is the influence of a one-on-one procedure announcement delivered by a radiation therapist before radiation therapy? In this trial randomizing 126 patients, no significant differences in mean trait or state anxiety scores measured before CT scan simulation, during the first and second sessions, or at the completion of radiotherapy were noted. Patients who benefited from the procedure announcement were significantly better informed of the treatment positioning and in vivo dosimetry. For patients, this specific procedure was not able to decrease their level of anxiety.

**Abstract:**

Background: Anxiety impacts patient outcomes and quality of life in response to cancer diagnosis. A prospective phase 3 trial randomizing 126 patients was conducted to determine whether a specific one-on-one procedure announcement provided by a radiation therapist before CT scan simulation decreases anxiety for patients with breast cancer requiring radiotherapy. Material and Methods: Anxiety was measured using the STAI form, and the QLQ-C30 and BR-23 questionnaires were used to evaluate quality of life. Results: Mean trait or state anxiety scores before CT scan simulation, before the first and second sessions, and at the end of radiation treatment were not significantly different. We observed a decrease in the level of anxiety with time; however, no significant difference in mean state anxiety scores at any of the time intervals was detected. Factors, such as anxiety trait score, professional and marital status, age, and use of alternative therapy, did not significantly influence the evolution of anxiety status over time or the mean value. Anxiety was significantly influenced by the level of fatigue. Patients who benefited from the radiation therapists’ advice felt significantly better informed. Conclusions: The one-on-one program announcement occurring before CT scan simulation led to patients being more informed and greater satisfaction but did not decrease anxiety.

## 1. Introduction

The procedure announcement is one of the quality measures of the French Cancer Plan 2003–2007 and is required for the authorization given to health care institutions to treat cancer patients [1]. Creating a procedure announcement and expanding the use of multidisciplinary treatment planning meetings are widely recognized as measures that have improved the quality of cancer care in terms of information quality, listening, and support. The procedure announcement responds to a request from patients. Specifically, a consultation delivered by an oncology nurse was created. However, despite this procedure announcement and taking into account the specificity of radiation treatment, providing specific information on the time before radiation treatment may be useful. Indeed, the evolution of radiotherapy has been so important in recent years that it is often difficult for oncology nurses, such as for non-radiation oncologist physicians, to provide specific, complete, and updated information. Moreover, radiation may inspire some fears, which can be limited by precise information provided by professionals who are directly in contact with the patient during treatment. Therefore, information delivered by a radiation therapist may improve the quality of information before radiation treatment [2]. This information session would offer the opportunity to reformulate some information provided by the radiation oncologist at the first visit, such as length of treatment, location of the dosimetric CT scan, dosimetry, and control during radiotherapy sessions. Furthermore, these professionals can complement some more practical information or advice. However, this complementary information needs to be evaluated to determine the potential psychological consequences and the impact on quality of life. The literature suggests that approximately 35% of all patients diagnosed with cancer experience emotional distress [3] with an anxiety rate of 40% for patients undergoing radiotherapy [4,5]. Among psychological responses to cancer diagnosis, anxiety is a feeling that is often reported and is becoming a field of interest as it directly impacts the patient’s outcome and quality of life [6].

We propose to evaluate the impact of such a procedure announcement on anxiety through a prospective randomized monocentric trial comparing two groups of patients who would receive or not receive a specific procedure announcement provided by a radiation therapist before radiation treatment for breast cancer.

## 2. Materials and Methods

This study was a 2-arm, randomized study designed to evaluate the effect of a specific procedure announcement provided by a radiation therapist before radiation therapy on anxiety in patients with breast cancer. This study was completed at a single institution: Centre Paul Strauss, ICANS, Strasbourg, France. This study was approved by our institutional review and registered at EudraCT N° ID-RCB: 2014-A000527-40. Written informed consent was obtained from all participants.

### 2.1. Patient Selection Criteria

Patients eligible for enrollment in this study were adults (age ≥ 18 years) with histological evidence of primary breast cancer who needed to undergo radiation treatment with curative intent. Inclusion criteria included a WHO performance status of 0 or 1 and the ability to read and converse freely in French. Patients were excluded if they had previously undergone radiotherapy, if they presented metastases and benefited from palliative treatment, or if they were affected by a severe psychological disorder preventing a good comprehension of the inventory, such as by a nervous breakdown. Patients were also required to provide valid informed consent prior to participation. All patients received an information booklet about the treatment procedure (CT scan simulation, positioning), side-effects, and advice at the time of first consultation with the radiation oncologist.

### 2.2. Randomization

**Figure 1:**
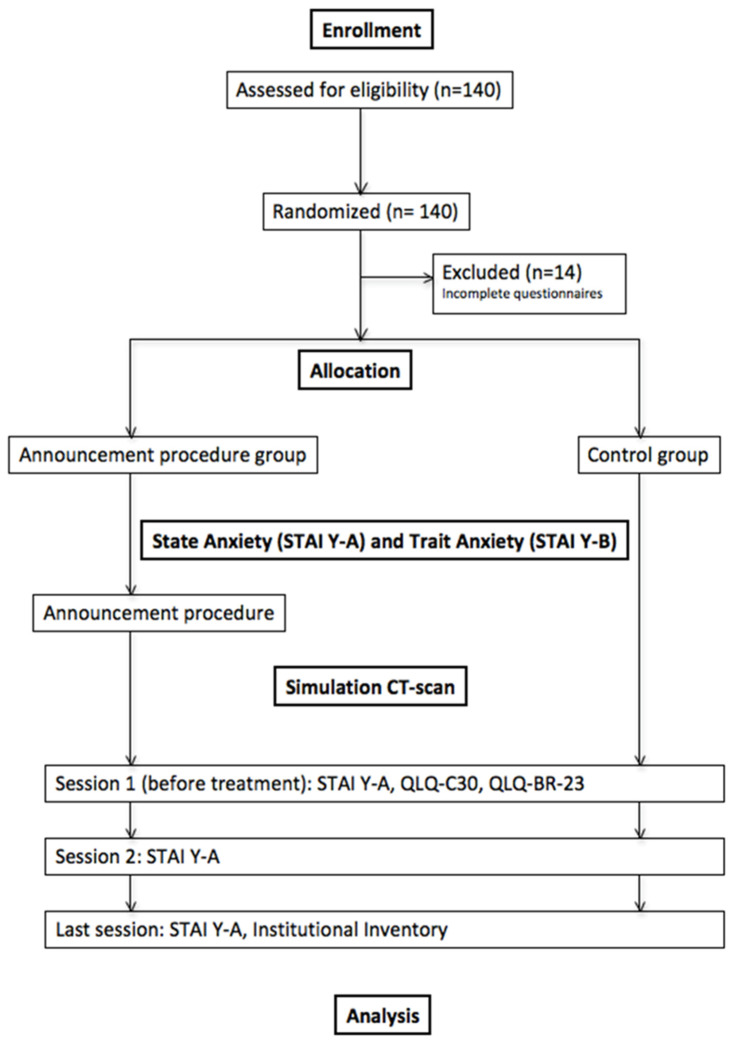
Trial design. Abbreviations: STAI Y-A = State Anxiety Inventory; STAI Y-B = Trait Anxiety Inventory; QLQ-C30 = EORTC (European Organization for Research and Treatment of Cancer) core quality of life questionnaire; QLQ-BR-23 = EORTC Quality of Life Questionnaire—Breast Cancer Module.

After the first consultation with the radiation oncologist, patients were randomly assigned to either a specific procedure announcement before the simulation CT scan or not. A statistician and a Clinical Research Associate (CRA) performed randomization. The trial design is shown in Figure 1.

### 2.3. Study Evaluation

The primary endpoint was the comparison of the evolution of anxiety evaluated at different times during radiation treatment between a group of patients treated according to the current procedures and a group of patients who would have benefited additionally from a standardized procedure announcement provided by a radiation therapist before the CT-scan simulation. The current state of anxiety was evaluated by the State Trait Anxiety Inventory—Y–A/B [7]. Secondary endpoints included evaluation of quality of life (EORTC QLQ-C30 and BR-23) and opinions of the patients about information provided (institutional inventory).

### 2.4. Procedure Announcement

A radiation therapist provided the procedure announcement for 30 min. A specific PowerPoint describing the machine that will be used to treat the patient is presented. Radiation therapists were supervised and trained by psychologists and radiation oncologists during the preparation of the procedure announcement and the power point. The procedure is divided into three parts: reception of the patient, treatment planning, and procedure of treatment sessions.

- **Reception of the patient:**
  - Identity check (first name, last name, birth date)
  - Radiation therapists’ presentation (first name and function)
  - If the patient wishes it, a third person can come with him
  - Explanation of the purpose of this procedure announcement
  - Evaluation of knowledge and information held after the medical consultation and delivery of the notebook of information
- **Treatment planning:**
  - Treatment schedule
  - Procedure of the simulation CT scan (patient’s position, devices to ensure the exactly same position every day, temporary skin marks or tattoos, pictures)
  - Determination of the exact area that will be treated by the radiation oncologist
  - Determination of the total radiation dose distribution by the staff working with the radiation oncologist (including physicists and dosimetrists)
- **Procedure of treatment sessions:**
  - Opening hours of the radiation department, number of treatment sessions, and treatment schedule
  - Rules of identito-vigilance, photo ID of the patient
  - Show the machine delivering external-beam radiation therapy called a linear accelerator
  - Explain the presence of microphones and cameras
  - In vivo dosimetry
  - Medical follow-up during the treatment
  - Reexplanations of potential side-effects of radiation therapy given by the radiation oncologist and what to do if it occurs
  - Information about transportation and its coverage, check of the address and phone number of the patient
  - Check machines and computer programs to ensure that the machine will give the correct dose of radiation to the appropriate area of the patient’s body
  - Ensure that the patient receives a notebook of information
  - Provide the institutional inventory of self-assessment to the patient and inform him that it has to be completed before the first session of radiotherapy
  - Information about ways to meet with other professionals, such as a psychologist, dietitian, or social workers, for pain care, if necessary
  - The date of the first treatment session will be noted on the patient’s appointment card
  - Answer the patient’s questions
  - Accompany the patient up to the waiting room and machine and show him where to place the appointment card to announce his arrival to the LINAC team.

### 2.5. Anxiety Evaluation and Quality of Life

Anxiety evaluation was performed at baseline before the procedure announcement with the radiation therapist using the State Trait Anxiety Inventory (STAI Y-A and STAI Y-B) [7]. The patients randomized in the standard arm will answer the same inventory before the CT scan simulation. The State Trait Anxiety Inventory is a questionnaire that allows us to study the expression of different types of anxiety within individuals and to measure anxiety levels in cancer patients. The STAI Y-A is designed to specifically assess current anxiety as opposed to baseline trait anxiety (STAI Y-B), which corresponds to an individual’s predisposition to anxiety determined by personality. The STAI consists of 40 questions, 20 of which are related to the STAI Y-A and 20 to the STAI Y-B.

Before the first and second sessions of treatment, all patients will answer the STAI Y-A again. Finally, at the end of treatment, all patients will again answer the STAI Y-A as well as the institutional questionnaire. The QLQ-C30 and BR-23 questionnaires measured quality of life at baseline (before the first session of treatment).

### 2.6. Institutional Inventory

The institutional inventory was composed of 43 questions (Table S1). There were three possible answers: yes/no/do not know. It was completed at the end of the radiation treatment.

### 2.7. Statistical Design and Analysis

#### 2.7.1. Sample Size

The total state anxiety score ranges from 20 to 80, and a higher value corresponds to a higher level of anxiety. For women with breast cancer, the mean state anxiety score was approximately 40. A variation from 5 to 10 units is clinically relevant. Preliminary measures of the difference in mean state anxiety for the same patient at different times of radiotherapy allowed us to estimate a standard deviation of 10 points. The aim was for a sample size of 64 patients in each arm (128 patients in all) based on a 5% type I error and 80% power with a standard deviation of 10 to detect a 5- to 10-point difference in the mean state anxiety score between the two arms. We planned to enroll 140 patients in total with 70 in each arm. Statistical analysis was performed at the completion of the study.

#### 2.7.2. Statistical Analysis

The difference in the mean state anxiety score at each time point compared with baseline was calculated. Student’s t-test and the Wilcoxon Mann-Whitney test were used to compare the two groups. Multiple linear regression analysis was performed to examine the predictive value of characteristics, such as time, trait, age, professional and marital status, use of alternative therapy, and fatigue. The Wilcoxon test was used to compare the mean QLQC-30 and BR-23 scores. Regarding the socioeconomic and institutional questionnaire, the chi-square test was used to compare the two groups. Analyses were performed with SAS statistical software (V9.3), SAS Institute, Inc., Cary, NC, USA.

## 3. Results

The baseline characteristics were equally balanced between the two different arms (Table 1). One hundred and twenty-six patients completed the study and were evaluable for analysis. During the course of the study, eight patients decided to stop the completion of questionnaires, and six patients requested to be excluded from the study.

### 3.1. Anxiety

For the Trait Anxiety Inventory (STAI Y-B), the difference in the mean value between the two arms was not significant (*p* = 0.95) (Table 2). Additionally, no significant difference between the two arms in the mean scores of state anxiety (STAI Y-A) measured at the different times of treatment (*p* = 0.32, *p* = 0.69, *p* = 0.85, and *p* = 0.41, at baseline, before the first and second sessions and at the completion of treatment, respectively) was noted between the two groups.

This study aimed to evaluate the evolution of state anxiety during treatment. Therefore, the differences in scores at the different times were measured and compared to the baseline score. The anxiety levels decreased significantly with time; indeed, the reduction between the end of treatment and before treatment was 6.0 and 7.86, respectively, for the control group and the experimental group (*p* < 0.0001). The effect of time was not significant between the two groups. When we measured the difference in the mean value at each time point for each group, we observed that the difference in the mean value was less than three points for the STAI. This difference was not clinically significant; a difference of 5 to 10 points was considered clinically relevant. The difference in the mean value between the two arms for the different times was not significant (*p* = 0.29, 0.40, and 0.60 before the first and second sessions and at the end of treatment, respectively).

The effects of the trait anxiety score, age, professional situation, marital status, use of alternative therapy, and fatigue on anxiety were also analyzed. Linear regression analyses were performed for the two parameters of the trait anxiety score and age. Five different professional situations were distinguished: working, not working, student, unable to work, and unemployed. Additionally, for marital status, six different situations were distinguished: married, widowed, divorced, single, living together, and civil partnership. The evolution of anxiety over time did not significantly depend on the trait anxiety score for either the control group or the experimental group. The test “between subjects” shows that the mean value of the state anxiety score during treatment depends significantly on the trait anxiety score (*p* < 0.001). Professional and marital status, age, and use of alternative therapy did not significantly impact the evolution of state anxiety over time or the mean value. The level of fatigue was explored with the QLQC-30 inventory. For the question ”Were you tired?”, patients could provide four different responses: not at all, a little, quite a bit, or very much. Fatigue significantly influenced the level of anxiety (Table 3). The more patients felt tired, the more anxious they were. The mean State Anxiety score was 27.21 for patients who gave the answer “not at all” tired, whereas the scores were 36.69, 36.55, and 35.58 for patients who felt “a little”, “quite a bit”, and “very much” tired, respectively (*p* < 0.0001). However, the response did not influence the impact of time for State Anxiety.

### 3.2. Quality of Life

The evaluation of quality of life has been achieved with the QLQC-30 and BR-23 inventories provided by the European Organisation for Research and Treatment of Cancer [8]. Compared to the mean EORTC scores, patients included in our study had a worse body image (*p* = 0.001) and better future perspectives (*p* = 0.0002) (Table 4). Fatigue and body image were significantly different in both arms. Patients who benefited from the procedure announcement were more tired (*p* = 0.02), had a worse perception of body image (*p* = 0.01), and had a worse future perspective (*p* = 0.03). The results of the State Anxiety and the QLQC-30 (some specific questions) were matched. Patients were classified into quartiles of state anxiety: patients who were less anxious had a better quality of life. Anxiety is directly related with quality of life.

### 3.3. Institutional Inventory

Ninety percent of patients who benefited from the procedure announcement judged it useful. Eighty percent of patients judged the book about radiation treatment useful; there was no difference between the two arms. Thirty-six percent of patients searched the Internet, and no difference was noted between the two groups. Patients who benefited from the procedure announcement were better informed than patients in the control group regarding position treatment (92% vs. 73.3%, *p* = 0.007, respectively), in vivo dosimetry (29.5% vs. 50.79%, *p* = 0.01, respectively), tattoos (87.1% vs. 78.7%, NS, respectively), and side-effects (80.9% vs. 68.8%, NS, respectively). Eighteen percent of patients requested that more information be provided (about machines, check, fatigue, length of treatment). None of the patients received information that they would not like to have. None of the patients would have preferred a shorter procedure announcement.

## 4. Discussion

The diagnosis of cancer and subsequent treatments lead to anxiety. Anxiety is a symptom that should not be underdiagnosed and underestimated because it directly negatively impacts quality of life and is associated with increased fatigue and negatively correlated treatment outcomes [9,10,11]. Different factors can be related to anxiety in patients with breast cancer, including physical, psychological, social, and environmental factors, such as age, side-effects of treatment, hormonal changes, perception about change in body image, social support, or visits to the hospital. Cox et al. and Jacobsen et al. examined anxiety in women with breast cancer receiving chemotherapy [12,13]. In the two studies, age and trait anxiety were identified as being predictive of anxiety during chemotherapy as well as two years after diagnosis. Several studies have shown that anxiety is high in patients treated by surgery but is independent of the type of surgery [14,15,16,17]. Wallace et al. assessed the effect of radiotherapy regimen schedules on the level of anxiety and did not show any significant differences in the level of anxiety between the short regimen and long regimen treatment schedules both before initiation and after completion of radiotherapy [18]. Schreier and Williams compared the level of anxiety in women with breast cancer who underwent radiation or chemotherapy for early-stage breast cancer [19]. This longitudinal descriptive study, which used the Ferrans quality of life index and STAI for evaluation, showed that the level of anxiety (both state and trait anxiety) was increased in women who underwent chemotherapy compared to women who underwent radiotherapy. The levels were measured prior to the start of treatment and at 4 weeks, 12 weeks and one year after these respective treatments.

The effect of information and educational support on anxiety has not often been evaluated. Our study did not demonstrate any significant difference in anxiety with or without a specific procedure announcement provided before CT scan simulation by a radiation therapist. One explanation could be that women are already very well informed before treatment. Indeed, many sources for breast cancer patient education exist. Many different methods, including novel technologies, provide a proof of concept of improved patient knowledge regarding their disease-specific condition and management. Verbal information can be delivered in groups or in an individual face-to-face session. Written information, for example, delivering booklets, is also an efficient method for patients’ education. Visual information can also be delivered, especially in the field of radiation therapy, because it can be the object of erroneous representations and fears on behalf of the patients. In a randomized trial, Thomas et al. showed that anxiety could be reduced significantly with an educational video provided prior to chemotherapy and/or radiotherapy [20]. In another randomized trial, Hahn et al. made the same conclusion with the same educational tool [21]. In our study, we used a PowerPoint presentation, which is a visual form used to deliver information. Attai et al. recently demonstrated that patients’ diagnostic information increases and their anxiety decreases with participation in a Twitter social medial support group [22]. Our institution is a cancer center; thus, specific attention is given not only to patient pathology and treatments but also to their social and psychological well-being. Each patient receives his own individualized care plan. For example, in each waiting room (consultation, chemotherapy units, radiotherapy department), TV screens are available to inform patients about their course of care and treatment procedure, and a dedicated information area is provided. Therefore, this complete environment could increase the patient’s confidence and provide more access to information. In contrast, increased importance may be given to technical aspects of radiation treatment in a radiation department of a general hospital.

In our study, patients who received the radiotherapy preparatory intervention did not report a significantly noteworthy decrease in psychological distress from baseline to completion of radiotherapy compared with the standard of care group. Our results confirm those of Halkett et al. Specifically, anxiety levels did not change between time from referral to radiotherapy, prior to treatment planning and after starting treatment [23]. In this study, 31% of women had clinically relevant levels of anxiety prior to treatment planning and 26% after treatment planning. The 123 women who were enrolled in this study were evaluated using the hospital anxiety and depression scales (HADs). The choice of the tool to evaluate anxiety in our study has to be highlighted. In addition to the HAD scale, the STAI form has the advantage of being a quantitative tool to evaluate a feeling, which is rather subjective. The STAI questionnaire is a widely accepted self-reporting tool used to measure anxiety levels in cancer patients. Our results are also consistent with those of Zissiadis et al., who measured anxiety using the STAI form [24]. Their conclusion was that more written information and a phone call provided by a nurse did not significantly change patients’ anxiety scores or satisfaction levels.

For patients included in the study from Halkett et al., written and verbal information by health professionals was the most preferred information source, and patients would have appreciated a one-on-one session with a radiation therapist [23]. Other randomized trials failed to show a significant reduction in anxiety with one-on-one encounters [25,26,27]. In our study, information was provided with a one-on-one session occurring before CT scan simulation, which is a very appropriate moment. Indeed, in a longitudinal study including 213 patients with breast cancer, Lewis et al. measured anxiety before and after the radiotherapy simulation and the first and last five radiotherapy sessions using visual analog scales (VASs) [28]. Patients were particularly anxious at the radiotherapy simulation and the first radiotherapy session. Anxiety decreased rapidly and dramatically at subsequent sessions during the first week of radiotherapy. Additionally, in a randomized controlled trial, D’Haese et al. showed that an additional educational intervention with written materials on the third or fourth day of treatment reduced patient anxiety compared with the provision of all information on the first visit [29]. Additionally, in 2018, Halkett et al. evaluated an RT preparation intervention consisting of face-to-face consultation with a radiation therapist prior to planning and prior to treatment [30]. The primary outcome measure was psychological distress using the total score for the HAD scale (HADs-T), which adds anxiety and depression scores together to provide a score for psychological distress. The authors demonstrated significantly lower HADs after the first day of treatment and second consultation with the radiation therapist. In contrast, before planning (and after the first consultation with radiation therapist) and at the end of treatment, no significant difference was noted. Additionally, concerning anxiety, significantly lower levels of anxiety after the first day of treatment when the treatment was completed were observed for patients who benefited from the two consultations, indicating that this is an appropriate time point for both radiation therapist-delivered interventions. Youens et al. provided the same reduced HAD scores at treatment commencement after two face-to-face consultations with a radiation therapist: prior to treatment planning and prior to the first day of treatment [31].

However, our study failed to demonstrate a significant difference in terms of anxiety, and the importance of standardized procedures for radiation therapists has to be highlighted. Often, the radiation therapist delivers some information once the patient is already on the table treatment. This may not be the appropriate moment because the patient may be unable to be open and to understand information. Moreover, it might be too late to respond appropriately to eventual emotional distress. The role of the radiation therapist is also very important; indeed, patients may be less likely to ask some questions to a radiation therapist than to a doctor. Additionally, this process provides the opportunity to make some psychosocial referrals. Braeken et al. performed a study evaluating a screening inventory of psychosocial problems in 268 cancer patients receiving curative radiotherapy treatment [32]. Thirty-three patients were proposed to a psychosocial care provider before radiation treatment, of whom 31 patients experienced subclinical or clinical psychosocial symptoms. Twenty-one patients were referred to a psychologist or social worker at this time point.

Although patients from the experimental group were significantly better informed, especially regarding tattoos, position treatment, and side-effects, they were not less anxious. This finding explains why other factors may be related to anxiety.

Anxiety is probably more related to fatigue than to information and educational support. Indeed, in our study, fatigue was a significant factor of anxiety. There is a relationship between cancer-related fatigue and anxiety and depression; however, the underlying mechanisms are unclear [33]. Our findings are consistent with those of the study of Ho et al. including 133 women with early-stage breast cancer. Specifically, anxiety was significantly associated with cancer-related fatigue in a multivariate analysis as well as perceived stress and pain severity [34]. The correlation between fatigue and anxiety is high, but the effect of this relationship remains uncertain. Does a cancer patient become anxious because of fatigue or might it be the reverse? Or both? Most studies have explored the relationship between depression and cancer-related fatigue. In many studies that explored both symptoms, depression and anxiety have often been considered one entity. However, our study was not designed to evaluate the relationship between anxiety and fatigue as a primary endpoint, which could be consistent with future studies. Additionally, future directions need to focus on long-term follow-up; it would be interesting to continue to follow the patients enrolled in our study and to appreciate the evolution of anxiety months and years after treatment because patients are often more anxious about lifestyle issues than treatment itself. Moreover, specific works are needed for specific radiation techniques or using specific medical devices, such as deep inspiration breath hold to reduce irradiated heart volume in breast cancer patients. In addition to information or knowledge, educational training programs delivered by radiation therapists should be evaluated.

This study has some limitations. The trial may have been underpowered to detect changes in quality of life and opinions of the patients about information provided given that the primary endpoint was changes in levels of anxiety evaluated by the State Trait Anxiety Inventory. Some patients were excluded from the study due to uncompleted inventories; however, this may not have had some impact on the findings reported because a potential drop-out rate was initially considered in the statistical design.

## 5. Conclusions

Although our study did not identify a significant difference in terms of anxiety, the procedure announcement provided by a radiation therapist should not be cancelled from the patient care plan because it provides the opportunity to detect other concerns or issues and to orient patients to appropriate health professionals, such as psychologists or social workers. Therefore, providing information can be meaningful given numerous other possible positive outcomes.

## 6. Implications for Practice

This randomized study revealed that radiation therapists are strongly implicated in patient care before radiation therapy. A specific procedure announcement before CT scan simulation allows better information, knowledge, and satisfaction levels for patients requiring radiation treatment.

## Figures and Tables

**Table 1:** Patient characteristics.

	Control Group (n = 61)	Experimental Group (n = 65)	Total (n = 126)	*p*-Value
Age (y) Median (range)	61 (28–82)	60 (33–81)	60 (28–82)	0.55
Karnofsky Index Median (range)	100 (80–100)	100 (80–100)	100 (80–100)	0.17
Psychologist (%) Yes No	10 (16.9) 49 (83.1)	10 (15.4) 55 (84.6)	20 (16.1) 104 (83.9)	1.00
Psychiatrist (%) Yes No	3 (5) 57 (95)	2 (3.2) 61 (96.8)	5 (4.1) 118 (95.9)	0.67
Anxiolytics (%) Yes No	11 (18.3) 49 (81.7)	9 (14.3) 54 (85.7)	20 (16.3) 103 (83.7)	0.63
Antidepressants (%) Yes No	8 (13.1) 53 (86.9)	4 (6.1) 61 (93.9)	12 (9.5) 114 (90.5)	0.23
Alternative therapy (%) Yes No	18 (29.5) 43 (70.5)	18 (29.5) 43 (70.5)	36 (29.5) 86 (70.5)	1.00
Marital status (%) Married Widowed Living together Divorced Civil partnership Single	41 (67.2) 4 (6.6) 2 (3.3) 4 (6.5) 0 (0) 10 (16.4)	37 (57.8) 7 (10.9) 2 (3.1) 12 (18.8) 2 (3.1) 4 (6.3)	78 (62.4) 11 (8.8) 4 (3.2) 16 (12.8) 2 (1.6) 14 (11.2)	0.07
Children (%) Yes No	51 (83.6) 10 (16.4)	61 (93.8) 4 (6.2)	112 (88.9) 14 (11.1)	0.09
Number of children (%) 1 2 3 4 5 6	10 (20.8) 25 (52.1) 10 (20.8) 3 (6.3) 0 0	17 (27.9) 24 (39.3) 12 (19.7) 5 (8.2) 2 (3.3) 1 (1.6)	27 (24.8) 49 (44.9) 22 (20.2) 8 (7.3) 2 (1.9) 1 (0.9)	0.65
Live (%) Alone With family With other adults	11 (18.0) 49 (80.3) 1 (1.7)	16 (25) 47 (73.4) 1 (1.6)	27 (21.6) 96 (76.8) 2 (1.6)	0.69
House (%) Rural area Urban area	37 (61.7) 23 (38.3)	38 (60.3) 25 (39.7)	75 (61.0) 48 (39.0)	1.0
Diploma (%) None Secondary education High school diploma Higher education	7 (11.9) 24 (40.7) 11 (18.6) 17 (28.8)	8 (12.5) 34 (53.1) 7 (11) 15 (23.4)	15 (12.2) 58 (47.2) 18 (14.6) 32 (26.0)	0.53
Professional status (%) Working Not working Pensioned Unemployed Unable to work	24 (39.4) 1 (1.6) 33 (54.1) 3 (4.9) 0 (0)	32 (49.3) 3 (4.6) 28 (43.1) 1 (1.5) 1 (1.5)	56 (44.4) 4 (3.2) 61 (48.4) 4 (3.2) 1 (0.8)	0.35
Professional category (%) Senior executive Intermediate Employee Worker Unemployed Craftsman	11 (18.6) 6 (10.2) 34 (57.6) 5 (8.5) 2 (3.4) 1 (1.7)	9 (13.8) 3 (4.6) 38 (58.5) 7 (10.8) 3 (4.6) 5 (7.7)	20 (16.1) 9 (7.3) 72 (58.1) 12 (9.7) 5 (4.0) 6 (4.8)	0.52

**Table 2:** Evaluation of Trait Anxiety (STAI Y-B) and State Anxiety (STAI Y-A) scores. STAI Y-A0: baseline score; STAI Y-A1: before first session of radiotherapy; STAI Y-A2: before second session of radiotherapy; STAI Y-A3: at completion of radiotherapy; Diff1a0: difference in mean STAI Y-A scores between baseline and first session of radiotherapy; Diff2a0: difference in mean STAI Y-A scores between baseline time and second session of radiotherapy; Diff3a0: difference in mean STAI Y-A scores between baseline time and completion of radiotherapy.

	Control Group (n = 61)			Experimental Group (n = 65)			Total (n = 126)			
	Mean	(95% CI)	Median	Mean	(95% CI)	Median	Mean	(95% CI)	Median	*p*-Value
**STAI Y-B**	37.15	34.83–39.47	37.00	37.14	34.91–39.37	37.00	37.14	35.56–38.73	37.00	0.95
**STAI Y-A0**	36.10	33.19–39.01	34.00	38.26	35.17–41.35	36.00	37.21	35.11–39.32	35.00	0.32
**STAI Y-A1**	36.08	32.88–39.29	33.00	36.61	33.62–39.60	35.00	36.35	34.19–38.51	34.00	0.69
**STAI Y-A2**	32.34	29.54–35.15	30.00	32.88	30.06–35.69	30.50	32.62	30.66–34.58	30.00	0.85
**STAI Y-A3**	30.10	27.08–33.12	26.00	30.46	27.98–32.94	28.00	30.28	28.36–32.20	27.00	0.42
**Diff1a0**	−0.02	−2.15–2.12	−1.00	−1.90	−4.09–0.28	−2.00	−0.97	−2.48–0.55	−1.00	0.29
**Diff2a0**	−3.75	−5.59–−1.92	−4.00	−5.56	−8.04–−3.09	−4.50	−4.68	−6.22–−3.14	−4.00	0.40
**Diff3a0**	−6.00	−8.75–−3.25	−6.00	−7.86	−10.68–−5.03	−6.00	−6.94	−8.90–−4.99	−6.00	0.60

**Table 3:** Influence of the level of fatigue explored with the QLQC-30 inventory on the level of anxiety.

Been Tired	n	STAI Y-A0 (Mean)	STAI Y-A1 (Mean)	STAI Y-A2 (Mean)	STAI Y-A3 (Mean)	Mean STAI (Mean)	*p*-Value
Not at all	33	30.31	29.55	25.22	23.81	27.21	<0.0001
A little	66	40.18	38.63	35.34	32.63	36.69	
Quite a bit	20	39.00	39.56	35.58	32.00	36.55	
Very much	7	41.67	38.17	33.17	29.33	35.58	

**Table 4:** Evaluation of quality of life using QLQC-30 and BR-23 inventories: fatigue, body image, future perspective.

QLQC-30/BR-23	Control Group (n = 61)	Experimental Group (n = 65)		Total (n = 126)	EORTC Scores	
	Mean	Mean	*p*-Value	Mean	Mean	*p*-Value
Fatigue	55.67	69.98	0.02	30.44	33.3	> 0.05
Body image	69.49	54.62	0.01	74.21	82.7	0.001
Future perspective	68.51	55.58	0.03	58.27	47.3	0.0002

## Data Availability

The data presented in this study are available on request from the corresponding author.

## Supplementary Materials

The following are available online at https://www.mdpi.com/article/10.3390/cancers13112572/s1, Table S1: Institutional inventory.
