# Supplementary material for: Anxiety during Radiation Therapy: A Prospective Randomized Controlled Trial Evaluating a Specific One-on-One Procedure Announcement Provided by a Radiation Therapist
Source: Cancers (Basel). 2021 May 24;13(11):2572. doi: 10.3390/cancers13112572 (PMC8197250; doi:10.3390/cancers13112572)
Supplement: Supplementary file 1 [file cancers-13-02572-s001.zip › cancers-1176653-supplementary.pdf]

Table S1. Institutional inventory. The institutional inventory was composed of 43 questions. There were three possible answers: yes/no/do not know (Table S1). It was completed at the end of the radiation treatment.

1) Did you receive a booklet about radiation therapy?
2) Who gave it to you? radiation oncologist, resident, radiation therapist, other (precise)
3) Did you consult the welcome booklet of the cancer center?
4) If yes, did you read it alone?
5) Or with someone?
6) Did you consult the specific area information of the cancer center?
7) Did you consult the Internet?
8) Did you discuss your disease with other patients?
9) Did you discuss the radiation treatment with your house doctor?
10) Did you discuss radiation treatment with another healthcare professional?
11) Did you know that you would undergo a CT scan before radiation therapy?
12) Who informed you about this point? radiation oncologist, resident, radiation therapist, other (be precise)
13) Did anyone talk you about treatment positioning?
14) Who informed you about this point? radiation oncologist, resident, radiation therapist, other (be precise)
15) Did anyone talk you about tatoos?
16) Who informed you about this point? radiation oncologist, resident, radiation therapist, other (be precise)
17) Did you know that you were under surveillance during radiation session?
18) Who informed you about this point? radiation oncologist, resident, radiation therapist, other (be precise)
19)Did anyone talk you about control positioning?
20)Who informed you about this point? radiation oncologist, resident, radiation therapist, other (be precise)
21) Did anyone talk you about dosimetry *in vivo*?
22) Who informed you about this point? radiation oncologist, resident, radiation therapist, other (be precise)
23) Did anyone talk you about side effects?
24) Who informed you about this point? radiation oncologist, resident, radiation therapist, other (be precise)
25) Did anyone talk you about the organization of transport?
26) Who informed you about this point? radiation oncologist, resident, radiation therapist, other (be precise)
27) Did anyone talk you about consultations during treatment?
28) Who informed you about this point? radiation oncologist, resident, radiation therapist, other (be precise)
29) Did anyone talk you about opening hours of the radiation department?
30) Who informed you about this point? radiation oncologist, resident, radiation therapist, other (be precise)
31) Did anyone talk you about the psychologist?
32) Who informed you about this point? radiation oncologist, resident, radiation therapist, other (be precise)
33) Did anyone talk to you about the pain management specialist?
34) Who informed you about this point? radiation oncologist, resident, radiation therapist, other (be precise)
35) Did anyone talk you about alternative therapy?
36) Did you use an alternative therapy?
37) In your opinion, do you think that the specific procedure announcement provided by a radiation therapist was useful?
38) Is there any information that you would have liked to receive and that you did not receive during this specific procedure announcement? What information?
39) Was there any information provided during this specific procedure announcement that you would have preferred not to know? What information?
40) Do you think that this specific procedure announcement should be scheduled at another time? If yes, what would be in your opinion the ideal moment?
41) Do you think that this specific procedure announcement should be shorter?
42) Do you think that the booklet about radiotherapy that you received was useful?
43) Do you think that other information should be in the booklet? Which?
